# High Affinity Humanized Antibodies without Making Hybridomas; Immunization Paired with Mammalian Cell Display and *In Vitro* Somatic Hypermutation

**DOI:** 10.1371/journal.pone.0049458

**Published:** 2012-11-14

**Authors:** Audrey D. McConnell, Minjee Do, Tamlyn Y. Neben, Vladimir Spasojevic, Josh MacLaren, Andy P. Chen, Laurence Altobell, John L. Macomber, Ashley D. Berkebile, Robert A. Horlick, Peter M. Bowers, David J. King

**Affiliations:** AnaptysBio, Inc., San Diego, California, United States of America; National Institute on Aging, United States of America

## Abstract

A method has been developed for the rapid generation of high-affinity humanized antibodies from immunized animals without the need to make conventional hybridomas. Rearranged IgH D(J) regions were amplified from the spleen and lymph tissue of mice immunized with the human complement protein C5, fused with a limited repertoire of human germline heavy chain V-genes to form intact humanized heavy chains, and paired with a human light chain library. Completed heavy and light chains were assembled for mammalian cell surface display and transfected into HEK 293 cells co-expressing activation-induced cytidine deaminase (AID). Numerous clones were isolated by fluorescence-activated cell sorting, and affinity maturation, initiated by AID, resulted in the rapid evolution of high affinity, functional antibodies. This approach enables the efficient sampling of an immune repertoire and the direct selection and maturation of high-affinity, humanized IgGs.

## Introduction

Recombinant antibodies represent the fastest growing class of new medicines, and generation of antibodies that meet specific criteria is increasingly important for therapeutic applications. The majority of therapeutic antibodies now available were derived from rodent immunization and the subsequent generation of a panel of hybridomas [1], [2]. Immunization of wild-type animals was used to produce the first therapeutic antibody, the anti-CD3 monoclonal antibody (mAb) muromonab [3]. Patients treated with muromonab, however, mounted an immune response that attenuated the half-life and resulting efficacy of the molecule. In an effort to address unwanted immunogenicity, numerous structure- and library-based strategies have been pursued for the humanization of murine antibodies, replacing constant regions and some or all of the non-specificity determining residues with corresponding human antibody sequence [4]. In addition, immunization of transgenic mice in which endogenous immunoglobulin (Ig) loci have been replaced by a repertoire of human heavy and light chain germline transgenes, followed by the generation of human Ig-producing hybridomas, has recently emerged as an effective way of generating human antibodies to many antigens [5]–[8]. Currently, more than 40 fully human antibodies produced in transgenic mice have advanced to clinical evaluation (reviewed in [9]).

Despite these innovations, novel approaches to therapeutic antibody development are still needed to address limitations associated with wild-type and transgenic animal immunization methods. The generation and screening of hybridomas from an immunized animal is time-consuming and samples only a fraction of the antibodies generated during the adaptive immune response. In addition, mAb humanization can be challenging when heavy and light chain variable (V) regions have limited homology to the closest human V-region, requiring iterative rounds of engineering and maturation to reach design metrics. Even when humanization is successful, the resulting antibody may still trigger an immune response. An approach that minimizes the non-human sequence incorporated into the final antibody without sacrificing functionality would therefore be useful.

Display methods for producing lead antibody candidates include phage, yeast, and mammalian display, and are based on the *in vitro* selection of antibody constructs from prepared libraries [10]. Such libraries provide a limited diversity that does not evolve *in situ* in the context of antigen specificity. While overcoming some of the limitations of the *in vivo* immune response, static libraries are challenged to generate high affinity antibodies and therefore rely on subsequent saturation mutagenesis-based affinity maturation [11]. Meanwhile, phage and yeast derived antibody fragments often require re-formatting to produce soluble, well-expressed antibodies with properties compatible with efficient manufacture and therapeutic utility [12], [13]. Mammalian cell display systems offer a number of potential advantages for therapeutic antibody generation, including the ability to co-select for key manufacturing-related properties such as high expression level and stability.

Here a method is demonstrated for generating high affinity humanized antibodies that pairs the flexibility of mammalian display and *in vitro* somatic hypermutation (SHM) with the robustness of the *in vivo* adaptive immune response. We applied this method to the selection and affinity maturation of functional antibodies to the human complement protein C5, a protein drug target for a number of therapeutic indications. C5 initiates formation of the membrane attack complex (MAC) in conjunction with proteins C6 and C7, a function of the innate immune response that targets foreign or damaged cells for lysis and elimination (reviewed in [14]). Inappropriate complement activation, MAC formation, and the resulting inflammatory response have been associated with a number of disease states such as paroxysmal nocturnal hemoglobinuria (PNH), uveoretinitis, atypical hemolytic uremic syndrome (aHUS), and osteoarthritis [15]–[17]. We sought to generate high affinity antibodies to C5 that would specifically inhibit MAC formation as potential human therapeutics for these and related indications.

In this study we aimed to isolate rearranged murine antibody sequences capable of binding a fragment of human complement protein C5. The CDR3 of the heavy chain (HCDR3) is a major determinant of antibody specificity [18], and is the antigen binding loop with the highest degree of diversity in both sequence and length. HCDR3 is encoded through the recombined D and J genes, which undergo both trimming and nucleotide addition during the recombination process [19]. IgH D(J) regions were amplified from spleen and draining lymph nodes of immunized mice. This diversity was combined with a limited repertoire of human heavy chain (HC) germline sequence V-genes, and paired with a human light chain (LC) library composed of several germline V-regions joined to diverse rearranged J-regions isolated from peripheral blood mononuclear cells (PBMCs) from a panel of normal human donors. Complete HCs and LCs were assembled with full-length constant regions supporting mammalian cell surface display, and transfected into HEK 293 cells co-expressing activation-induced cytidine deaminase (AID) to initiate somatic hypermutation (SHM) *in vitro*. Numerous C5-specific clones were isolated by fluorescence-activated cell sorting (FACS), and iterative rounds of SHM resulted in the identification of high affinity antibodies capable of blocking complement fixation. This approach enables the direct selection and maturation of high-affinity, humanized IgGs with superior pharmaceutical properties and minimal non-human sequence content.

## Results

### Generation of a humanized antibody library from immunized mice

**Figure 1:**
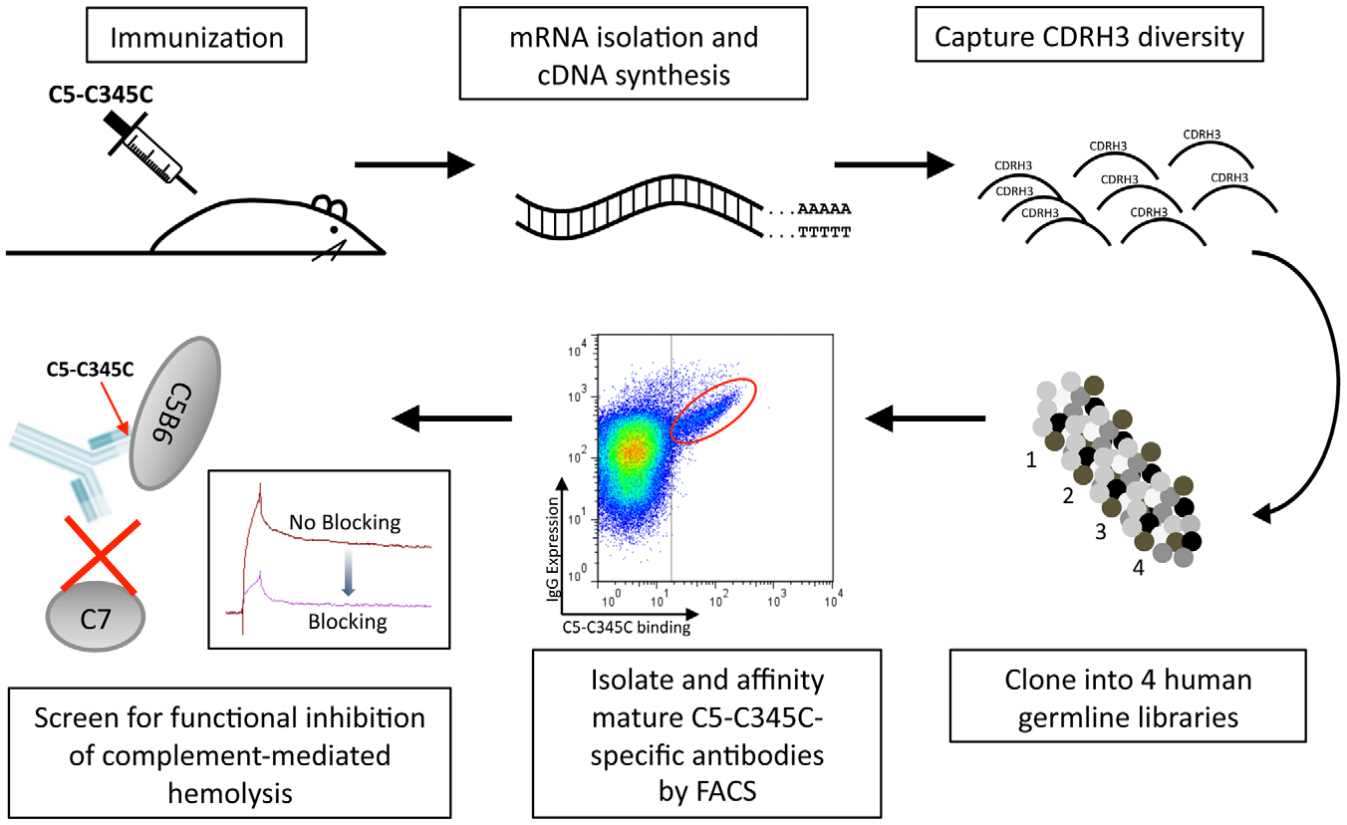
Isolation of high-affinity monoclonal antibodies from immunized mice. Spleen and draining lymph nodes were harvested from mice immunized with the C345C fragment of complement protein C5. After RNA isolation and cDNA synthesis, (D)J diversity was amplified from HCs and cloned into nine human germline variable regions (IGHV 1-2, 1-69, 3-7, 3-23, 3-30, 4-34, 4-59, 5-51, and 6-1). These were paired with a fully human LC library, composed of five germline IGκV regions (IGKV 1-33, 1D-39, 2D-30, 3-20, and 4-1), combined with CDR3 diversity obtained from a pool of normal human donors. Libraries were transfected into HEK 293 c18 cells as four sub-library pools for cell surface display and FACS selection of antigen specific clones. Subsequent affinity maturation by AID-induced mutagenesis and FACS resulted in a high-affinity antibody with demonstrated functional activity.

To generate humanized anti-C5 antibodies, mice were immunized with a small domain, C345C, comprising the C-terminal 147 residues of the 1648 residue C5 protein, that has been shown experimentally to initiate MAC formation via contacts with proteins C6 and C7 [20]. Immunoglobulin heavy chain D(J) regions were amplified from cDNA derived from spleen and lymph nodes of immunized mice, and cloned into an episomal vector containing the IgG1 constant region linked to a transmembrane sequence for cell surface display and a small repertoire of nine fully human germline V-regions, combined into four sub-libraries (Figure 1). Each HC sub-library was transfected and stably selected together with a LC library composed of five human germline kappa V-regions fused to J-region sequences isolated from pooled PBMCs from normal human donors as described previously [21]. Alternative heavy chain splicing allowed for the simultaneous surface display and secretion of full-length antibodies. Germline V-genes for the HC and LC libraries were selected based on the highest observed frequency of usage *in vivo* [22], [23].

### Isolation of human antibodies to C5-C345C

**Figure 2:**
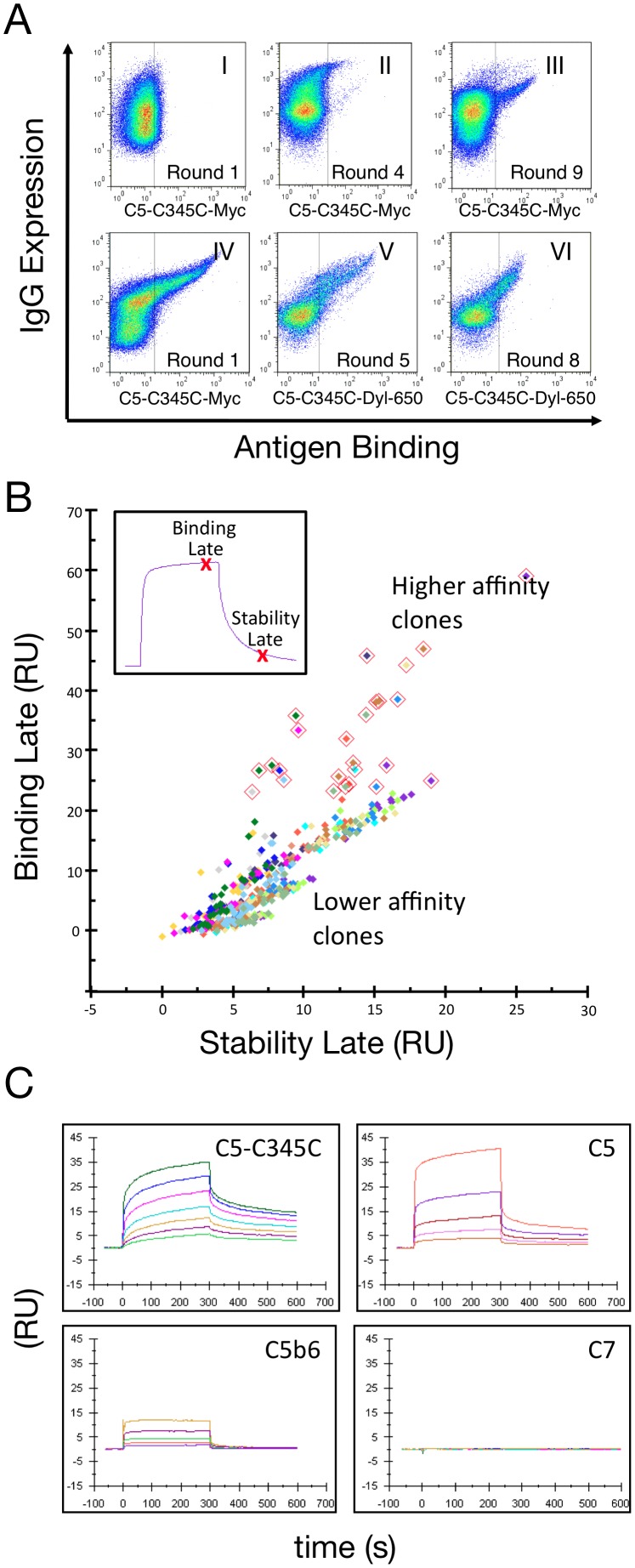
Discovery and affinity maturation of C5-C345C-specific antibodies. (*A*) FACS scattergrams depicting isolation of a C5-C345C-specific population from the sub-library pool containing IGHV3-30-3 and IGHV4-34 (panels I–III), and subsequent affinity maturation of APE777 in strategy B (panels IV–VI). Approximately 5×10^7^ cells were sorted per round. IgG expression is shown on the y-axis and C5-C345C binding on the x-axis. (I) The first sort round (5 µM C5-C345C-Myc) showed little detectable binding; (II) round 4 (100 nM C5-C345C-Myc); and (III) round 9 (3 nM C5-C345C-Myc) which included a room-temperature incubation to increase stringency. Affinity maturation FACS scattergrams of isolated C5-C345C-specific antibody, APE777, are shown for strategy B starting with (IV) round 1 (20 nM C5-C345C-Myc), (V) round 5 (1.5 nM C5-C345C-Dyl-650), and (VI) round 8 (100 pM C5-C345C-Dyl-650). Each sort round consisted of AID-induced SHM and selection of highest antigen binding and antibody expressing cells by FACS. Note emergence of new cell populations that exhibited increased antigen binding relative to the original population over time, and which were able to recognize sequentially decreasing antigen concentrations. (*B*) Plot comparing stability late (value corresponds to amount of antibody-bound antigen) versus binding late (value corresponds to off-rate, k_d_) capture-adjusted report points for Biacore 4000 screen of single cell clone supernatants. Higher values correspond to more antigen bound per unit antibody (binding late) and slower off-rates (stability late). Highlighted points indicate highest affinity C5-C345C binding clones further characterized by Biacore T200 and sequencing. (*C*) Full kinetic analysis of APE777 binding to C5-C345C (first panel) with a K_D_ of 200 nM (k_a_ = 1.4×10^5^ M^−1^ s^−1^, k_d_ = 2.4×10^−2^ s^−1^). Binding to C5 and C5b6 complement proteins was detectible (second and third panels), while APE777 shows no significant binding to the C7 negative control.

After selecting for stable episomal expression, the four sub-libraries were each expanded and subjected to iterative rounds of AID-induced mutation and selection by FACS using multimerized, fluorescently labeled antigen to create high avidity binding conditions. Selective pressure for high level expression of IgG was maintained by 2-dimensional sorting with both fluorescently labeled antigen and anti-human IgG labeled with a complementary fluorescent dye. By the fourth round of sorting, an emerging cell population was identified that expressed antibodies binding to C5-C345C from sub-library 3, derived from constructs using V-regions IGHV3-30-3 and IGHV4-34 (compare Figure 2a panels I and II). Sorts were continued with increased stringency by decreasing the concentration of C5-C345C antigen used to stain cells prior to each round of FACS. A corresponding enrichment of cell populations exhibiting improved C5-C345C binding appeared at each round of FACS (Figure 2a, panels I–III).

To identify antigen specific HC/LC pairs capable of binding complement protein C5, single cell clones from this population were isolated by FACS into 96-well plates, and the binding kinetics for each characterized by Biacore. Secreted antibody from the cell growth media was captured on the surface of a CM5 chip containing immobilized anti-human Fc antibody, with varying concentrations of C5-C345C analyte presented. Report points for each sensorgram identified antibodies with the most antigen bound per unit antibody (binding late) and slowest off-rates (stability late), indicative of better binding (Figure 2b). Of the 176 clones screened, supernatants from 25 of the highest affinity clones were further characterized by Biacore, and 18 of the strongest binders were sequenced to identify eight unique HC/LC pairs. The same antibody HC and LC sequences observed in these clones were also enriched after the final round of FACS sorting of sub-library 3 (see Figure 2a, panel VI). These included three HCs, each in the IGHV3-30-3 framework with unique CDRH3 sequences, and four LCs, two in the IGKV1-33 framework and two in the IGKV3-20 framework; each with a unique CDRL3 sequence. Isolated clones were analyzed based on binding kinetics for C5 domain C5-C345C, binding to the full-length C5 protein, binding to the activated C5bC6 complex, and absence of binding to a C7 negative control. Clone APE777 was found to bind C5-C345C with an affinity of approximately 200 nM, to bind C5 with approximately 10-fold reduced affinity, and showed minimal binding to activated C5b6. Binding to C7 was undetectable as expected (Figure 2c). APE777 was the only clone that exhibited detectable binding to the C5-C345C antigen in the absence of avid binding conditions. Based on these criteria, APE777 was selected for further affinity maturation.

### Affinity maturation

The isolated HC/LC pair from APE777 was transfected together with AID for *in vitro* affinity maturation by somatic hypermutation in the mammalian cell display system described above. Two independent cell cultures were affinity matured in parallel, designated as strategies “A” and “B.” Following induction of somatic hypermutation via expression of AID, cells expressing higher affinity variants of the starting antibody were isolated by iterative rounds of FACS sorting using decreasing concentrations of fluorescently labeled C5-C345C, with approximately 0.5% of the brightest cells collected at each round. Early rounds of sorting for each strategy were carried out at low nM concentrations of C5-C345C under avid binding conditions (Figure 2a, panel IV). Starting in round three, antibody affinities for C5-C345C in each strategy were sufficiently improved to use directly labeled monovalent antigen without binding avidity (C5-C345C-Dylight-650; see Figure 2a, panels V and VI). Affinity maturation was first observed in FACS scatter plots by the third and fourth rounds of selection for both strategies, and continued through round eight using a final concentration of 100 pM C5-C345C (Figure 2a, panel VI).

**Figure 3:**
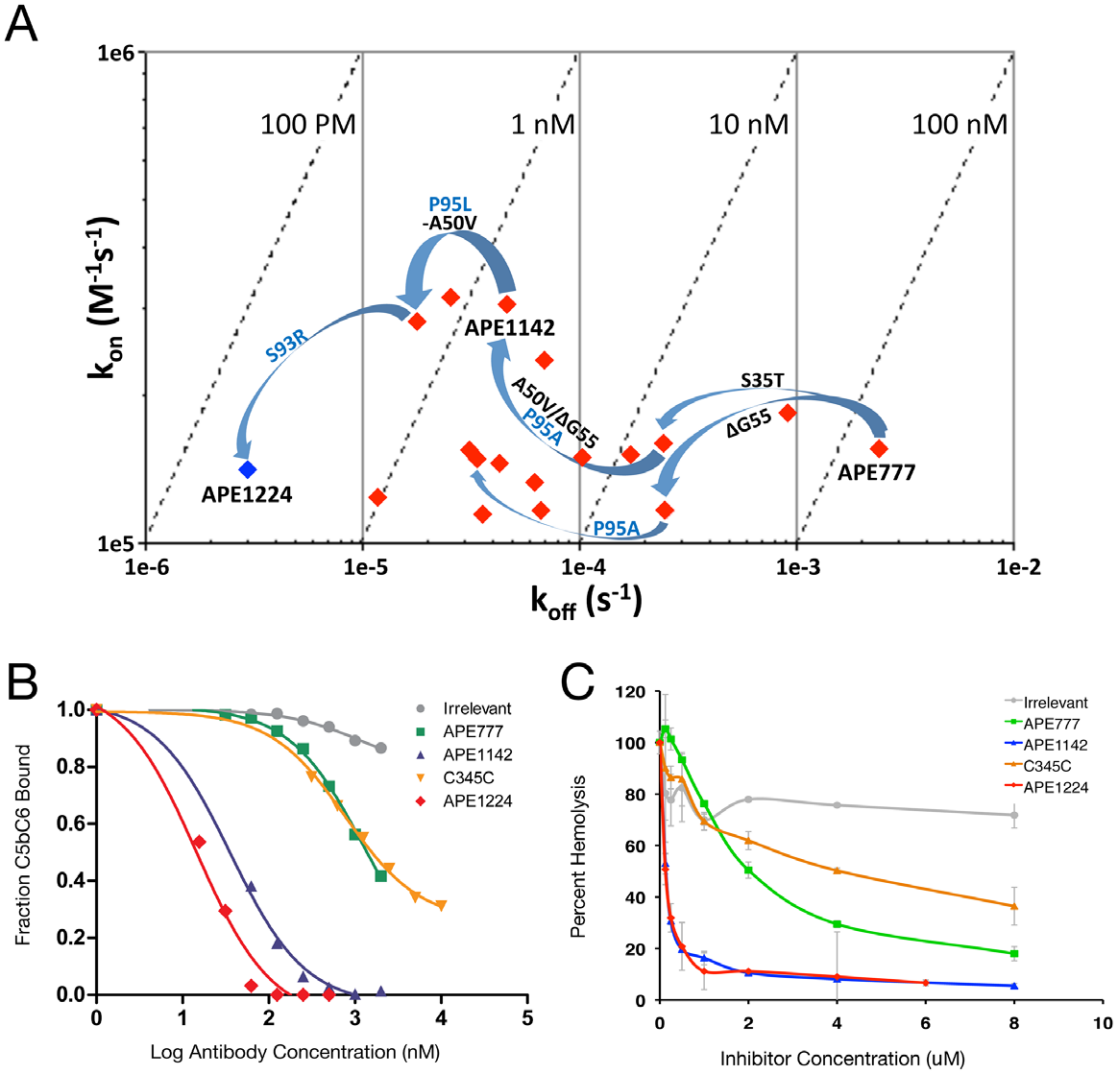
Kinetic and functional characterization of isolated C5-C345C-specific antibodies. (*A*) Plot of on-rate (k_a_) versus off-rate (k_d_) depicting anti-C5-C345C clones throughout the affinity maturation process as determined by Biacore T200 analysis. Iterative rounds of AID-induced mutagenesis and SHM resulted in mutations that improved the K_D_ 1000-fold from the starting antibody (APE777) with the majority of improvement obtained by a reduction in k_d_. Key mutation events are indicated by blue arrows with HC mutations indicated in black and LC mutations indicated in blue. Note that arrows represent affinity progression of improved antigen binding as independent mutations resulting from SHM were recombined and tested in different antibody contexts; changes shown do not represent phylogenetic relationships. (*B*) Summary of Biacore blocking assay demonstrating inhibition of MAC assembly, *in vitro*. Varying concentrations of antibody or control protein were incubated with a constant concentration (100 nM) of C5b6, and this was flowed over a CM5 surface with C7 captured via an anti-C7 antibody. Fraction C5b6 bound to C7 was calculated by comparing the maximum signal (RU) obtained in the presence and absence of inhibitor. (*C*) Hemolysis assay demonstrating inhibition of opsonized sheep red blood cell lysis mediated by the classical complement pathway. Graph depicts concentration of antibody, control isotype-matched antibody or control C5-C345C protein relative fraction red blood cell lysis using 1% serum. Percent specific lysis was normalized to lysis observed in the absence of added inhibitor, control antibody, or control protein.

Sample HCs and LCs from the evolving antibody populations were sequenced to identify enriching mutations over the course of affinity maturation in both strategies (Table 1). The majority of mutations were observed in the LC, beginning with the CDRL3 mutation, G92D, which enriched in the third round. Two HC mutations, S35T and A50V, were detected as enriched sequences in the fourth round of both strategies. A single residue deletion of G55 in the CDRH2 emerged in strategy B at round 4. LC mutations in strategy A included P95L, S93R, and a five amino acid insertion, QYGSS, at position 95 in the CDRL3. LC mutations in strategy B included P95A, S93I, and a six amino acid insertion, GSSPEY at position 97 in the CDRL3. Each of these enriching mutations arose independently in a single antibody context (Table 1). Mutations were subsequently combined to evaluate improvement in antigen binding. Biacore analysis was used to evaluate C5-C345C binding affinity of isolated and recombined clones throughout the maturation process (Figure 3a).

**Table 1:** Summary of observed enriching mutations.

Observed mutations	Chain	Starting sequence	Ending sequence	Start NA	End NA	Strategy	Enriched in round^1^
L18P	HC	CCTGA	CCCGA	t	c	A	4
S35T	HC	G**AGCT**	GACCT	g	c	A/B	4/5
A50V	HC	**AGCT**A	AGTTA	c	t	A/B	4/5
Delete G55	HC	T**GGTA**	T A	n/a	n/a	B	4
N100K	HC	**AACT** A	AAATA	c	a	B	5
S30I	LC	C**AGCA**	CATCA	g	t	A	8
G92D	LC	T**GGTA**	TGATA	g	a	B	3
S93R	LC	**AGCT**C	AGGTC	c	g	A	7
S93I	LC	T**AGCT**	TATCT	g	t	B	7
+QYGSS after S94	LC	TCACCG	TCACAGTATGGTAGCTCACCG	n/a	n/a	A	5
P95A	LC	CACCG	CAGCG	c	g	B	4
P95L	LC	ACCGG	ACTGG	c	t	A	5
P95T	LC	CACCG	CAACG	c	a	B	5
+GGSPEY after Y96	LC	**TACA**CT	TACGGTGGTAGCTCACCGGAGTACACT	n/a	n/a	B	7

Table of HC and LC point mutation, insertion, and deletion events observed during affinity maturation of APE777. The first column indicates the amino acid mutation, listed with Kabat numbering. The second column indicates whether the mutation is in the HC or LC. Columns three and four show the starting and ending nucleotide sequence surrounding the site of mutation, with the mutated nucleotides underlined. Columns five and six indicate the starting and ending nucleotide for point mutations. The last two columns indicate which strategy the mutation was observed in, and at which round it was enriched. Ten of the 11 point mutations were initiated at G or C nucleotides; one of the 11 was initiated at a T. Seven of the point mutations, the deletion, and one of the insertions occurred at known AID hotspots (WRCH, highlighted in **bold**) located within CDRs 1, 2, or 3. Seven of the 11 point mutations were nucleotide transitions, and four were nucleotide transversions.

1 Enrichment observed by Sanger sequencing of 40 HC and LC variable regions post-sort round.

Mutations observed in both strategies were directly recombined by overlap extension PCR into a 576-member combinatorial library for transfection and expression in 96-well format. Biacore screening identified several clones with low nM affinity for C5-C345C, and one, APE1224, with a 200 pM binding affinity representing a 1000-fold improvement in K_D_ over APE777 (Figure 3a). APE1224 contained two HC mutations (S35T and a deletion of G55) and two LC mutations (S93R and P95L).

### Inhibition of MAC assembly and complement-mediated hemolysis

A Biacore-based blocking assay was developed to assess the functional activity of isolated and matured anti-C5-C345C antibodies. Formation of the membrane attack complex of complement involves proteolytic cleavage of C5 to C5a and C5b, followed by sequential binding of activated C5b to C6, C7, C8, and C9 proteins [14]. Antibodies targeting C5-C345C are expected to block binding of C5b6 to C7. In this assay, the C5b6 complex was incubated with varying concentrations of antibody variants (or C5-C345C used as positive control) and flowed over immobilized C7 for detection of binding (Figure 3b). The original antibody, APE777, demonstrated modest blocking activity with an inhibition constant, K_i_, of 41 nM, but did not completely inhibit the C5bC6/C7 interaction at a 2 µM concentration. The matured anti-C5-C345C variants APE1142 and APE1124 exhibited significantly improved blocking activity with K_i_ values of 930 pM and 370 pM, respectively.

To further illustrate functional activity, anti-C5-C345C antibody variants were tested for their ability to inhibit hemolysis of antibody-coated sheep erythrocytes mediated by the classical complement pathway. Inhibition of complement activation was measured at 1% serum concentration over a range of antibody concentrations (Figure 3c). Percent specific lysis was determined as the amount of erythrocyte lysis relative to lysis in the absence of inhibitor antibody or protein. Hemolysis was weakly inhibited by C5-C345C over the indicated concentration range. Anti-C5-C345C antibody APE777 reached complete inhibition at 8 µM, and was a stronger inhibitor than C5-C345C. The matured APE1142 and APE1224 variants were stronger inhibitors than APE777, both reaching complete inhibition of complement-mediated lysis in this assay at a concentration of 1 µM (Figure 3c).

## Discussion

In this study we demonstrate an alternative approach to antibody discovery that combines mouse immunization with rapid humanization using mammalian cell surface display and AID-induced affinity maturation. This method obviates the need to isolate and screen individual hybridomas by generating a humanized antibody library directly from immunized mice. The rearranged heavy chain D(J) regions from mice immunized with the antigen C5-C345C antigen were PCR amplified from spleen and lymph node cDNA, and incorporated into synthesized germline IGHV regions. This HC library was combined with a human LC library [21], yielding humanized antibodies enriched for potential antigen binders. Transfection of this library into HEK 293 cells together with AID, and in a format compatible with surface display, facilitated the FACS selection of numerous C5-C345C specific antibodies. One of these clones was selected for subsequent affinity maturation to a K_D_ of 200 pM, and was demonstrated to possess a potent, dose-dependent ability to inhibit MAC formation and complement-mediated cell lysis.

This method couples the robust humoral adaptive immune response with maturation by AID-induced SHM, mimicking the critical features of adaptive immunity *in vitro* to produce high affinity antibodies. Incorporating HC D(J) regions from immunized mice into the displayed library provides greater potential to recover high affinity, efficacious antibodies for the antigen of interest relative to HC D(J) diversity derived from unselected, antigen-naïve sources. This approach also minimizes the amount of mouse sequence incorporated into the final antibody, in contrast to standard hybridoma and humanization strategies. We recovered eight unique HC/LC pairs from initial discovery, and anticipate that this method could be scaled to provide additional diversity. The subsequent application of mammalian display in combination with SHM allows selection pressure to be applied to evolving populations of antibodies such that they may be driven to desired end points including high affinity and specificity, and also other important parameters such as stability and high expression levels in mammalian cells. APE1224, composed of V-regions IGHV3-23 and IGKV3-20, was typical of antibodies isolated using this approach, expressing well in HEK 293 cells and unfolding with a *T*_m_ of 78°C, indicative of high thermostability. This method is potentially applicable to antibodies from any origin, and although demonstrated here through active immunization of mice, it may equally well be applied to harvesting antibodies from the immune repertoire of other species, including humans.

The use of antibodies containing germline V-sequences as a starting point for affinity maturation, coupled with mammalian display and *in vitro* somatic hypermutation, has several advantages for the isolation of therapeutic antibodies. Mutations can be recombined in order to optimize binding affinity and functional activity; the resulting variants can also be selected for other desired properties such as solubility, expression, and post-translational modification. This allows a minimal number of mutations to be selected for incorporation into the final antibody, thus minimizing the impact of affinity maturation on the immunogenicity of the final molecule; APE1224 achieved a 1000-fold improvement in K_D_ with the incorporation of four key mutations. The method recapitulates the non-random natural process evolved through mammalian phylogeny and eliminates the need for saturation mutagenesis, modeling, or other predictive approaches that guess key residues at the antigen-antibody interface. Future work will include a direct comparative study to further demonstrate the robustness of this method for antibody discovery and optimization relative to other approaches.

Reproducing AID-directed SHM *in vitro* expands the potential sequence space that can be sampled during affinity maturation; mutations are not limited to pre-defined positions and amino acids. Mutation patterns in this study were typical of AID-mediated mutagenesis, generating nucleotide transitions as well as transversions, with mutations generally localized to WRCH AID hotspots (Table 1). In addition to amino acid substitutions, duplications and deletions of residues were observed in each of the two affinity maturation strategies, and both types of mutation resulted in improved binding affinity to antigen. Such mutations, which are a known feature of *in vivo* SHM mediated by AID [24], would be difficult to predict or design into a library and further demonstrate the benefit of utilizing AID to induce mutagenesis.

These results demonstrate the flexibility of mammalian display and *in vitro* SHM to discover and evolve potent, neutralizing antibodies starting with only a minimal component of HCDR3 diversity. We have previously demonstrated that pairing human HCDR3 and LCDR3 diversity from a naïve repertoire with germline V-regions is sufficient as a starting point for discovery and affinity maturation [21]. V-gene sequences have been characterized for their conformational plasticity [25]. When paired with AID-mediated mutations, these sequences provide a vast repertoire of nucleation points for initially low-affinity binding and subsequent affinity mutation during both *in vivo* adaptive immunity and in our *in vitro* SHM system. The elasticity of this approach, leveraging low-affinity binding with avidity and continuously directed AID-mediated evolution, facilitates the development of antibodies with tailored properties.

The highest affinity antibody in this study, APE1224, demonstrated significant functional activity in both *in vitro* and *in vivo* assays measuring MAC assembly and the inhibition of complement-mediated hemolysis. APE1224 binds the C5-C345C domain of complement protein C5 with picomolar affinity and is able to block MAC assembly by inhibiting the interaction between C5b6 and C7, an essential step in the classical complement pathway. Improper regulation of MAC formation has been implicated in several human disorders. Eculizumab, targeting C5, is the only current pharmaceutic that blocks C5 activation and subsequent MAC formation, and has been approved for clinical use in PNH and aHUS indications. Eculizumab functions by binding C5 and blocking its cleavage by the C5 convertase at sites of complement activation, thereby inhibiting formation of both C5a, an anaphylatoxin, and C5b, which initiates MAC formation. We sought to develop an antibody that would specifically target C5b and MAC formation, thereby eliminating some the immune-suppressive related side-effects associated with eculizumab, while focusing efficacy on those indications most associated with inappropriate MAC assembly. Recent studies have additionally indicated that complement plays an important role in human osteoarthritis. C5 activation, and specifically MAC formation, was demonstrated to be crucial to the development of arthritis in three mouse models of the disease, and expression of inflammatory and degradative molecules was lower in chondrocytes from destabilized joints from C5-deficient mice [17]. An antibody targeting C5 to block MAC assembly, such as APE1224, could be an effective treatment for these and other indications.

## Materials and Methods

### Ethics statement

All animal handling work was conducted in accordance with national and international guidelines following the Guide for the Care and Use of Laboratory Animals. All research animal use was conducted according to protocols reviewed and approved by the Antibody Solutions Institutional Animal Care and Use Committee. The number of animals used in this study was minimized and all necessary precautions taken to mitigate pain or suffering.

### Immunization and tissue harvest

Immunization and tissue harvest procedures were carried out by Antibody Solutions (Sunnyvale, CA). Three female BALB/c mice aged 6–8 weeks were immunized with 0.005 mg of sterile-filtered C5-C345C in PBS together with alhydrogel/muramic dipeptide (ALD/MDP) adjuvant. The C345C-adjuvant mixture was injected into the foot pad of each mouse with a 26-gauge needle. Bi-weekly immunizations were evenly spaced over the course of 28 days. Mice were bled from the tail vein before injections and blood stored at −20°C for later analysis. Mice were euthanized, spleen and draining (popliteal) lymph nodes harvested, and cell pellets resuspended in RNAzol for further analysis (Sigma-Aldrich, St. Louis, MO).

### Preparation of CDRH3 fragments and library construction

Spleen and lymph node cells were lysed and total RNA isolated using the RNeasy RNA isolation kit according to the manufacturer's protocol (Qiagen, Valencia, CA). Reverse transcription from total RNA was performed by RT-PCR using a combination of random decamers and oligo(dT) primers and 2 ug total RNA according to the manufacturer's protocol of the RETROscript Kit (Ambion). Following cDNA synthesis, CDRH3 diversity was amplified from the cDNA by PCR using the primers listed in Table S1. PCR products were gel purified, and CDRH3 amplification was confirmed by TOPO-cloning and Sanger sequencing of the PCR products. This amplified CDRH3 diversity was then inserted into nine human HC germline V-regions (IGHV1-2, 1-69, 3-7, 3-23, 3-30-3, 4-34, 4-59, 5-51, and 6-1), selected based on their frequency of usage *in vivo*. The LC library consisted of five germline Vκ-regions (IGKV1-33, 1D-39, 2D-30, 3-20, and 4-1), also selected based on their *in vivo* usage frequency. Full-length IgGs were assembled using IgHC-γ1 and IgκC constant domains. The C-terminal end of the HC was modified with a transmembrane domain to enable cell surface expression. The HC library DNAs were pooled into four separate sub-libraries for transfection, each containing two to three germline IGHV templates.

### Library selection

To isolate C5-C345C-specific binders, each of the four HC sub-library pools was transfected in combination with the five pooled LC libraries into HEK 293 c18 cells, stably selected, and expanded to 1×10^9^ cells. Binding analysis was performed prior to each round of FACS in order to determine the optimal antigen concentration for selection. Antibody-transfected cells were incubated with various concentrations of C5-C345C-Myc for 0.5 h at 4°C. Dylight-649-labeled mouse anti-Myc (AbCam) was added at a 2∶1 (antigen∶anti-Myc antibody) molar ratio, and cells were incubated for 0.5 h at 4°C. To stain for IgG expression, FITC-AffiniPure Fab Fragment Goat anti-Human IgG (H+L) (Jackson ImmunoResearch) was added (1∶500) for 0.5 h at 4°C. Cells were pelleted and resuspended in 0.3 ml of DAPI (4′,6-diamidino-2-phenylindole dihydrochloride, Sigma-Aldrich); 0.2 µg/ml in PBS, 0.1% BSA; and analyzed for fluorescence on a BD Influx cell sorter (BD Biosciences). Antibody-expressing HEK 293 c18 cells (5×10^7^ in 20 ml PBS, 0.1% BSA) were incubated with a selected concentration of C5-C345C-Myc for 0.5 h at 4°C for cell sorting. Dylight-649-labeled mouse anti-Myc and FITC-labeled goat anti-human IgG Fc were added to the cells as described above. Cells were then resuspended in 1.0 ml DAPI (0.2 µg/mL in PBS, 0.1% BSA) and sorted for the strongest antigen-binding cells, relative to level of antibody expression, on a BD Influx cell sorter.

### Affinity maturation using mammalian cell display

Each stable HEK 293 c18 cell line episomally expressing an IgG HC modified with a C-terminal transmembrane domain for surface expression, together with an episomally expressed LC, was generated as described [21]. Antibody surface expression was confirmed by staining with FITC-labeled goat-anti-human IgG Fc (Jackson ImmunoResearch). IgG-expressing cell lines were stably transfected with plasmids encoding activation-induced cytidine deaminase (AID) for affinity maturation. To promote further mutagenesis, cells were additionally transiently transfected with an AID expression vector five days prior to each round of selection by FACS, as described above, using C5-C345C-Myc, mouse anti-Myc-Dylight-649, and FITC-goat anti-human IgG Fc staining. As affinity maturation progressed, later rounds of selection were performed without anti-Myc avidity using C5-C345C-Dylight-650 at decreasing concentrations.

### Protein expression and purification

A Myc-his-tagged variant of the C5-C345C antigen fragment of the human C5 protein was expressed from a pET15b plasmid in the *Origami B* strain of *Escherichia coli* following standard methods. Briefly, *E. coli* transfected with the C5-C345C expression plasmid was grown at 30°C, and expression induced with 1 mM isopropyl β-D-thiogalactoside (IPTG) at OD_600_ = 0.8. Upon induction, cultures were shifted to 18°C and shaken overnight. Cells were harvested by centrifugation and lysed with BugBuster reagent following the manufacturer's protocol (Novagen). Clarified lysate was purified by his-tag affinity purification and buffer exchanged into 10 nM Tris-HCl/150 mM NaCl, pH 7.5.

Human IgG antibody variants were produced using the QuickChange II site-directed mutagenesis kit (Agilent Technologies) or standard overlap/extension pcr techniques [26]. Full-length antibodies were expressed transiently in HEK 293 c18 cells, purified using a protein A/G agarose resin (Thermo Scientific), washed with 6 column-volumes of 1× PBS, pH 7.4, and eluted with 100 mM glycine, pH 3.0, followed by buffer exchange into 1× PBS, pH 7.4.

To screen combinations of HC and LC mutations derived from affinity maturation, HEK 293 c18 cells were transiently transfected with HC/LC pairs in 96-well array format, and antibodies characterized directly from supernatants.

### Affinity characterization of antibody/C5-C345C binding

Secreted antibodies from 96-well plate array transfections were screened and ranked using a Biacore 4000 (GE Healthcare). Each of four spots in four flow cells on a Series S CM5 chip (GE Healthcare) was coupled with ∼10,000 response units (RU) anti-human IgG (Fc) (Human Antibody Capture Kit, GE Healthcare). Culture medium from antibody transfected HEK293 cells was diluted 1∶10 with HBS-EP+ buffer (0.2 M Hepes, 3 M Sodium Chloride, 60 mM EDTA, 1.0% Polysorbate 20). Secreted antibody was captured on the CM5 chip by flowing diluted culture medium over the outer spots for 120 s at 10 µl/min. The captured surface was then washed with 1 M NaCl for 45 s at 30 µl/min, after which C5-C345C at 500 nM and 50 nM was passed over all flow cells for 120 sec at 30 µl/min, then allowed to dissociate for 300 s. The capture surface was regenerated using glycine, pH 2.0, for 120 s. Resulting sensorgrams were analyzed and ranked using Biacore 4000 Evaluation Software version 1.0.

Antibody variants, including those exhibiting the highest affinity and expression by Biacore 4000 analysis, were chosen for scale-up, purification, and additional characterization of kinetic constants using a Biacore T200 (GE Healthcare). A capture assay was used to allow accurate assessment of antibody affinity. Antibodies at 1 µg/ml were captured for 60 s at 10 µl/min on a Series S CM5 chip surface immobilized with approximately 3,000 RU anti-human IgG (Fc). C5-C345C antigen was flowed over the captured IgG surface using a range from 10-fold above to 10-fold below the K_D_ in each case. Surfaces were regenerated with 3 M MgCl_2_ for 180 sec. Association and dissociation kinetic constants (k_a_ and k_d_) were determined from a best fit o the data using the 1∶1 Langmuir global fitting procedure to sensorgrams in the Biacore T200 Evaluation Software version 1.0.

### Biacore blocking assay

The surface of a series S CM5 chip was immobilized with 3,000 RU of anti-C7 capture antibody (Complement Tech). Complement protein C7 at 100 µg/ml (Complement Tech) was captured for 60 s at 10 µl/min. Antibodies or C5-C345C, over a concentration range with 2-fold dilutions from 2 µM to 62.5 nM or 500 nM to 15.6 nM, as indicated, were incubated with a constant 100 nM concentration of C5b6 and then flowed over the captured C7 surface. Fraction C5b6 bound to C7 was then calculated by dividing the maximum signal (RU) for each sample by that obtained for C5b6 binding to C7 alone. To determine K_i_ values of antibody variants, normalized data were fit by a three-parameter inhibition curve using GraphPad Prism (GraphPad Software).

### Hemolysis blocking assay

Recombinant C5-C345, APE777 parental antibody, APE1142 and APE1224 affinity matured antibodies, or an isotype matched control antibody were diluted 2-fold in a round-bottom 96-well plate in the presence of 1% normal human serum (Complement Tech, #NHS). Rabbit antibody-sensitized sheep erythrocytes (1.25×10^7^ cells in 25 ul; Complement Tech, #B202) were added to each well and plates incubated at 37°C for 45 minutes on a plate shaker. Reactions were quenched with 100 µl ice cold GVB^++^ (0.1% gelatin, 5 mM Veronal, 145 mM NaCl, 0.025% NaN_3_, pH 7.3, 0.15 mM calcium chloride, 0.5 mM magnesium chloride; Complement Tech, #B102), plates centrifuged at 1250×g for 5 minutes, and supernatants assessed for hemoglobin release by reading OD_450_ (SpectraMax). All reagents were diluted in GVB^++^ buffer, and each sample was independently assayed in triplicate.

### Sanger sequencing

Standard Sanger sequencing of 40 heavy chains and light chain genes from sorted cells subsequent to each FACS round revealed enriching SHM-induced mutations.

## Supporting Information

Table S1 **List of primers used for amplification of CDRH3 diversity from immunized mice.** Uppercase nucleotides represent Eag I (CGGCCG) and Nhe I (GCTAGC) Type II restriction sites for cloning purposes. Lowercase nucleotides represent annealing sequences upstream of the CDRH3 (forward primers MMu.FP1-5) and in the HC constant region (reverse primers Mmu.RP1-3).
(DOCX)
