# Supplementary material for: High Affinity Humanized Antibodies without Making Hybridomas; Immunization Paired with Mammalian Cell Display and In Vitro Somatic Hypermutation
Source: PLoS One. 2012 Nov 14;7(11):e49458. doi: 10.1371/journal.pone.0049458 (PMC3498135; doi:10.1371/journal.pone.0049458)
Supplement: Table S1 — List of primers used for amplification of CDRH3 diversity from immunized mice. Uppercase nucleotides represent Eag I (CGGCCG) and Nhe I (GCTAGC) Type II restriction sites for cloning purposes. Lowercase nucleotides represent annealing sequences upstream of the CDRH3 (forward primers MMu.FP1-5) and in the HC constant region (reverse primers Mmu.RP1-3). (DOCX) [file pone.0049458.s001.docx]

| **Table S1.** List of primers used for amplification of CDRH3 diversity from immunized mice | |
| --- | --- |
| **Primer** | **Sequence** |
| Mmu.FP1 | CGGCCGtgtattactgtgcaaga |
| Mmu.FP2 | CGGCCGtgtattactgtgcgaaa |
| Mmu.FP3 | CGGCCGtgtattactgtgcgaga |
| Mmu.FP4 | CGGCCGtatattactgtgcgaaa |
| Mmu.FP5 | CGGCCGtgtattactgtgcgaga |
| Mmu.RP1 | GCTAGCgggaagacggatgggccctt |
| Mmu.RP2 | GCTAGCgggaagaccgatgggccctt |
| Mmu.RP3 | GCTAGCgggaaaagggttggggcgga |
